# TIGIT as a Novel Prognostic Marker for Immune Infiltration in Invasive Breast Cancer

**DOI:** 10.2174/1386207325666220629162823

**Published:** 2023

**Authors:** Chenming Guo, Zhiwen Luo, Dilimulati Ismtula, Xiaojuan Bi, Han Kong, Yiyang Wang, Zhen Yang, Xinmin Mao

**Affiliations:** 1 State Key Laboratory of Pathogenesis, Prevention and Treatment of High Incidence Diseases in Central Asia, The First Affiliated Hospital of Xinjiang Medical University, Urumqi, Xinjiang, 830011, China;; 2 Department of Breast Surgery, Center of Digestive and Vascular, The First Affiliated Hospital of Xinjiang Medical University, Urumqi, Xinjiang, 830011, China;; 3 College of Pharmacy, Xinjiang Medical University, Urumqi, Xinjiang, 830011, China

**Keywords:** TIGIT, invasive breast cancer, immune checkpoint, prognosis, bioinformatics analysis, tumor immunity

## Abstract

**Background:**

To assess the levels and potential therapeutic and prognostic significance of TIGIT in invasive breast cancer.

**Methods:**

The Cancer Genome Atlas database was used to evaluate TIGIT levels in invasive breast cancer and its association with clinicopathological features. Immunohistochemistry (IHC) was performed to validate it. Further, the Kaplan-Meier survival curve, univariate and multivariate Cox regression models were applied in analyzing the role of TIGIT in the prognosis of invasive breast cancer. Go / KEGG enrichment analyses techniques were used to investigate the possible cellular mechanism, and string database was used to explore TIGIT-related proteins. Finally, the TIMER database was used to determine the association between TIGIT and immune cell infiltrations.

**Results:**

TIGIT was differentially expressed in Pan cancer tissues compared with normal tissues. Relative to normal tissues, TIGIT levels in invasive breast cancer were elevated (*p<*0.05). TIGIT mRNA level was significantly different from T stage, age, ER and PR level (*p<*0.05). The high levels of TIGIT exhibited positive correlations with PFI and OS (*p<*0.05). Univariate analysis revealed that age, clinical stage, high TNM stage, menopausal status and radiotherapy were the factors affecting OS (*p<* 0.05). Multivariate analysis revealed that age, high clinical stage and menopausal status were independent risk factors for tumor progression (*p<*0.05). CD226, INPP5D, PVR, PVRL2 and PVRL3 proteins interact with TIGIT. The TIGIT levels were significantly correlated with infiltrations of immune cells (such as CD8^+^ T cells) (r=0.917, *p<*0.05).

**Conclusion:**

TIGIT is elevated in invasive breast tumor and is closely associated with the prognosis of invasive breast cancer. TIGIT may be the target of immunotherapy for invasive breast cancer.

## 1 INTRODUCTION

Advanced breast cancer is associated with an overall survival rate of 20% after 5-years of comprehensive treatment [1, 2]. The International Agency for Research on Cancer (IARC) reported that, in 2020, more than 2,260,000 new breast tumor cases and 685,000 deaths were recorded in China, which have replaced lung cancer as the most prevalent tumor globally. The breast cancer burden in China is rising, with the number of breast tumor cases in China accounting for 18.4% of the global breast cancer burden [3, 4]. Invasive breast cancer accounts for about 80% of all breast cancers. Due to a complex pathological classification system, unique biological microenvironment, and individual differences, breast cancer still cannot be cured. Breast cancer mortality is often due to metastasis and recurrence. Some breast tumor subtypes, including triple negative breast cancer (TNBC), are not responsive to endocrine and targeted therapies and currently, only 10-20% of TNBC patients benefit from immunosuppressive therapy targeting PD-1/PD-L1 [5]. Thus, there is an urgent need for novel immunosuppressive therapy.

TIGIT is an inhibitory receptor of the immunoglobulin family bearing immunoreceptor tyrosine-based inhibitory motifs (ITIM) [6]. TIGIT expression is tightly restricted to lymphocytes, such as CD8^+^ T, CD4^+^ T, and natural killer (NK) cells [7, 8]. TIGIT has a high affinity for its ligand CD155, which regulates the immune function of lymphocytes by interacting with the CD155 trans oligomer [9]. A recent study found that TIGIT is elevated on surfaces of tumor infiltrating lymphocytes and that CD155 is elevated on cancer cell surfaces. In mice, blocking the TIGIT/CD155 axis suppresses the progression of head and neck squamous cell carcinoma [10]. TIGIT is elevated in gastric cancer patients, where it may regulate the metabolic immune efficacy of CD8^+^ T cells, and the synergistic blockade of TIGIT and programmed death receptor-1 (PD-1) promoted the immune efficacy of CD8^+^ T cells [11]. Currently, a limited number of studies have examined the levels and prognostic significance of TIGIT in invasive breast cancer.

Here, seeking to identify new therapeutic targets, we assessed the levels and prognostic significance of TIGIT in invasive breast cancer. To this end, we analyzed TCGA datasets for the relationship between TIGIT levels in invasive breast cancer and its clinicopathological features. We then used immunohistochemistry to assess TIGIT expressions in breast tumor tissues. To elucidate the cellular mechanisms underlying TIGIT activity, we carried out GSEA database and determined the relationship between TIGIT and immune cell infiltration using the TIMER and GSVA packages on R (v3.6.3).

## 2 MATERIALS AND METHODS

### 2.1 Data Collection

Data on 1222 invasive breast cancer samples (113 paracancerous tissues and 1109 cancer tissues) were acquired from TCGA (https://portal.gdc.com) to analyze TIGIT in invasive breast cancer and its relationship with Progression-free Interval (PFI) and Overall Survival (OS) in invasive breast cancer patients. The KM-plotter database has data on mRNA levels and prognostic parameters. The suggested probes for candidate immune checkpoint genes were analyzed. Breast cancer cases were assigned into 2 groups based on median gene expressions. The relapse free survival (RFS), OS and log-rank P-value were determined.

### 2.2 Tissue Samples

The tissue samples were obtained from 9 patients with invasive breast cancer diagnosed by the breast surgery department of the First Affiliated Hospital of Xinjiang Medical University from Jan 2018 to Jun 2019. The breast cancer tissues were obtained by surgery and their matched para cancer tissues (≥5 cm from the tumor margin). All *patients were* women (mean *age*: 52±15.1 years; range: 28-72 years). The inclusion criteria were: (1) pathology-confirmed diagnosis of invasive breast cancer, (2) surgical resection (complete resection of primary tumor and regional lymph node dissection, with margins histologically confirmed to be free of cancer, and (3) availability of complete data on clinicopathology and follow-up. Exclusion criteria were: i. Presence of distant metastases, and ii. Anti-cancer therapy prior to surgical resection. *PFI* denoted the period from surgical date to disease progression or relapse (distant or local). *OS* was determined as the period from operation date until the date of last follow-up or death. *Patients without* an *event or death were* censored *at the* time of *last* known *follow-up* and *data* obtained *via* phone call *or* from *outpatient* records. From each patient, tumor/non-tumor tissue pairs were obtained. Ethical clearance for the study was granted by the institutional review board of the First Affiliated Hospital of Xinjiang Medical University. Participants were restaged using guidelines from the 8^th^ American Joint Committee on Cancer (AJCC) [12].

### 2.3 Immunohistochemistry

Immunohistochemistry on formalin-fixed, paraffin-embedded tissues was done by the Envision Detection System (DakoCytomation, Carpinteria, CA). After dewaxing and hydration, incubation of tissue sections in the presence of 3*%* H_2_O_2_ was done for 10 min to block endogenous peroxidase activities. Retrieval of the antigen was done by microwaving the samples in citrate buffer. The tissues were then blocked using 10% normal goat serum for 1 h before incubation with anti-TIGIT primary antibody (ab243903, Abcam, 1:200) at 4°C, overnight. Negative control samples were subjected to the same treatment, but without the primary antibody. They were then incubated at room temperature (RT) with secondary antibody (*1:500*) for 2h. Signal was then developed using freshly prepared diaminobenzidine (DAB), and sections lightly counterstained with hematoxylin. The resulting solution was then washed fully with water after differentiation till it turned blue; routine dehydration and transparence was performed followed by neutral gum mounting. They were then examined and imaged on a Leica DM 3000 microscope and analysis was done using ImageJ.

### 2.4 Protein-protein Interaction (PPI) and Functional Enrichments

To identify TIGIT-related proteins, a PPI network of TIGIT was constructed using STRING. Enrichments of TIGIT and related proteins were analyzed using Gene Ontology (GO) and Kyoto Encyclopedia of Genes and Genomes (KEGG) pathway analysis. Finally, we use Gene set enrichment analysis (GSEA) for verification.

### 2.5 Correlation Analyses of Immune Cell Infiltrations

The Tumor Immune Estimation Resource (TIMER) was used to assess the relationship between TIGIT and tumor purity, and various immunocytes, including B cells, CD4^+^ T cells neutrophils, macrophages, and CD8^+^ T cells. Correlations between TIGIT and infiltrations of immune cells were evaluated by Spearman correlation analysis.

### 2.6 Statistical Analysis

The *SPSS* software version 13.0. was used for analyses. Associations between TIGIT expression and clinicopathological variables were analyzed using the *χ^2^* test. Survival analysis was done using the log rank test and Kaplan-Meier method. Cox proportional hazards regression analysis was used to evaluate univariate and multivariate hazard ratios (HR) for PFI and OS. Univariate analysis variables with *p=*<0.05 were subjected to multivariate analyses for the selection of independent prognostic factors. The risk of individual factors was estimated using HR with 95% confidence interval (CI). Finally, R was used to establish a nomogram and build a prediction model. *p=*<0.05 indicated significant differences.

## 3 RESULTS

### 3.1 TIGIT Levels are Elevated in Invasive Breast Cancer

**Fig. (1):**
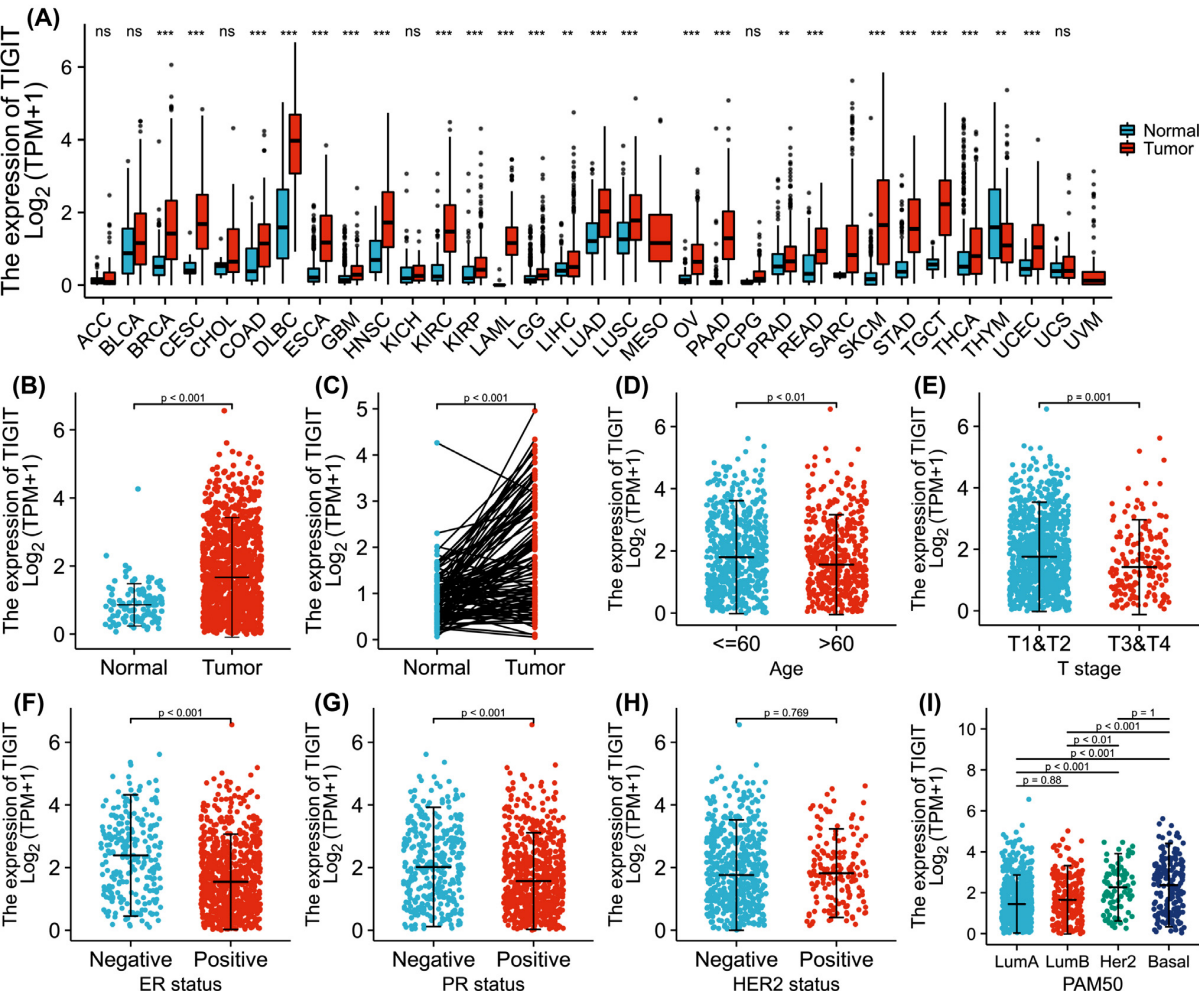
The levels of TIGIT in cancer. (**A**) relative to corresponding normal tissues, the level of TIGIT in different cancer tissues is different (*p<0.05); (**B**) The levels of TIGIT in invasive breast cancer were markedly high than in normal tissues (*p<*0.001); (**C**) In the paired samples, the levels of TIGIT in invasive breast cancer was significantly increased (*p<*0.001); (**D-I**) Association between TIGIT and clinical manifestations of invasive breast cancer.

**Fig. (2):**
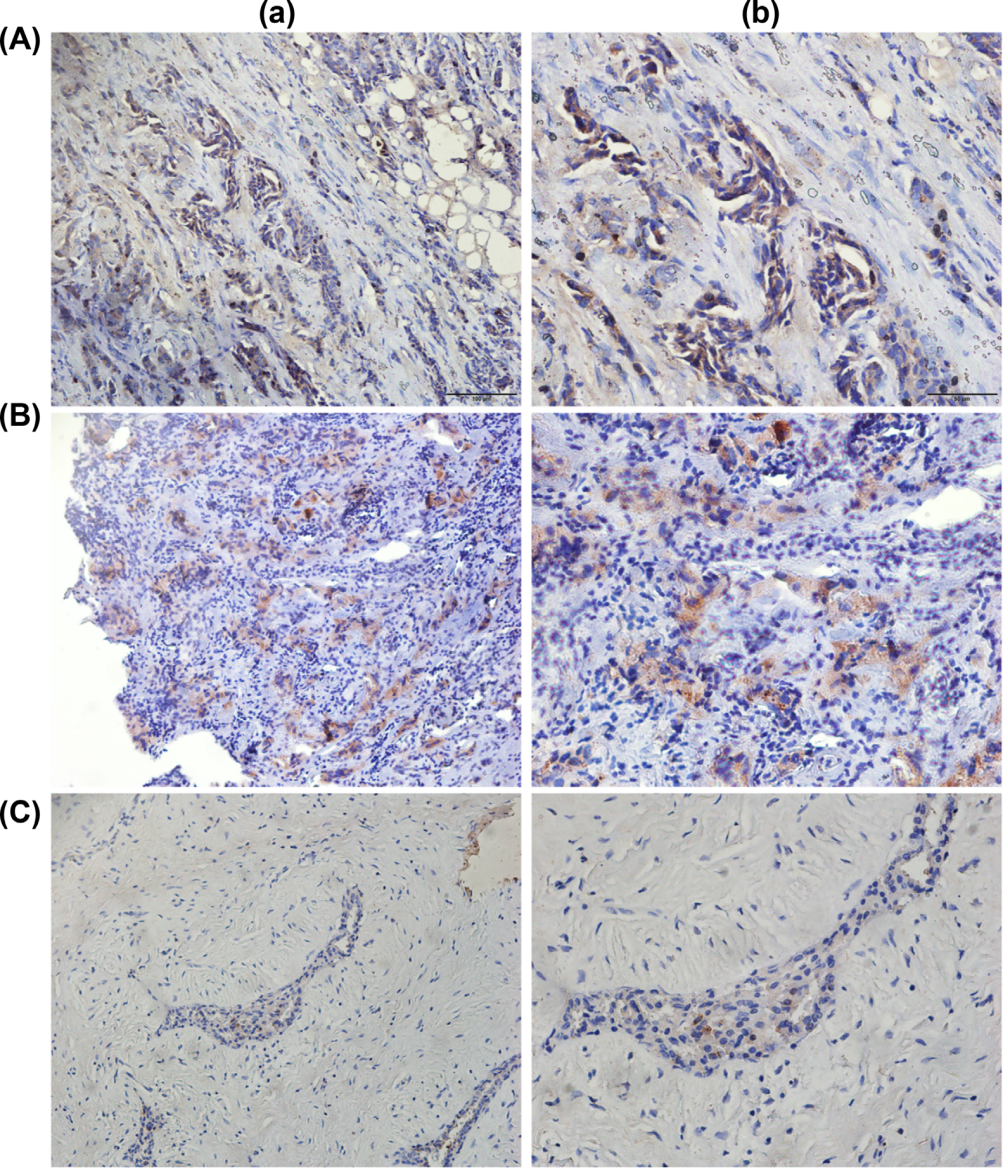
Protein expression level of TIGIT in invasive breast tumor tissues and their paired normal tissues. (**A-B**) Representative images of immunohistochemically stained invasive breast cancer tissues (a, magnification, ×200; b, magnification, ×400); (**C**) Representative images of immunohistochemically stained normal tissues (a, magnetism ×200; b,magnification ×400).

Analysis of differential TIGIT expression in pan cancer cells revealed elevated TIGIT mRNA levels in invasive breast cancer, cervical cancer, glioblastoma multiforme, colon cancer, diffuse large B cell lymphoma, esophageal cancer, head and neck squamous cell carcinoma, clear cell renal cell carcinoma, acute myeloid leukemia, brain low-grade glioma, renal papillary cell carcinoma, hepatocellular carcinoma, lung squamous cell carcinoma, lung adenocarcinoma, ovarian serous cystadenocarcinoma, pancreatic cancer, prostate cancer, rectal adenocarcinoma, gastric adenocarcinoma, cutaneous melanoma, testicular germ cell tumor, thyroid cancer, and endometrial cancer (Fig. **1A**). In paired and unpaired samples, TIGIT levels were significantly elevated in invasive breast tumor tissues relative to normal tissues (*p=*<0.001). Similar observations were made using IHC (Fig. **2**). Correlation analysis found that TIGIT had significant differences with age (*p=*<0.01), ER level (*p=*<0.001), T stage (*p=*0.001), and PR level (*p=*<0.001), but not with HER2 level (*p=*0.77). Relative to luminal A and B, TIGIT was significantly upregulated in HER2 overexpressing and basal type breast cancer (*p=*<0.01, Fig. **1B**-**I**).

### 3.2 Clinical Characteristics

Clinical histories of 1065 patients, including TNM stage, pathological stage, age, histological type, PR, ER, HER2, PAM50, menopausal status, and radiotherapy status, as well as OS, DSS, and PFI events were obtained from TCGA (Table **1**).

### 3.3 TIGIT Upregulation Correlates with Invasive Breast Cancer Prognosis

**Fig. (3):**
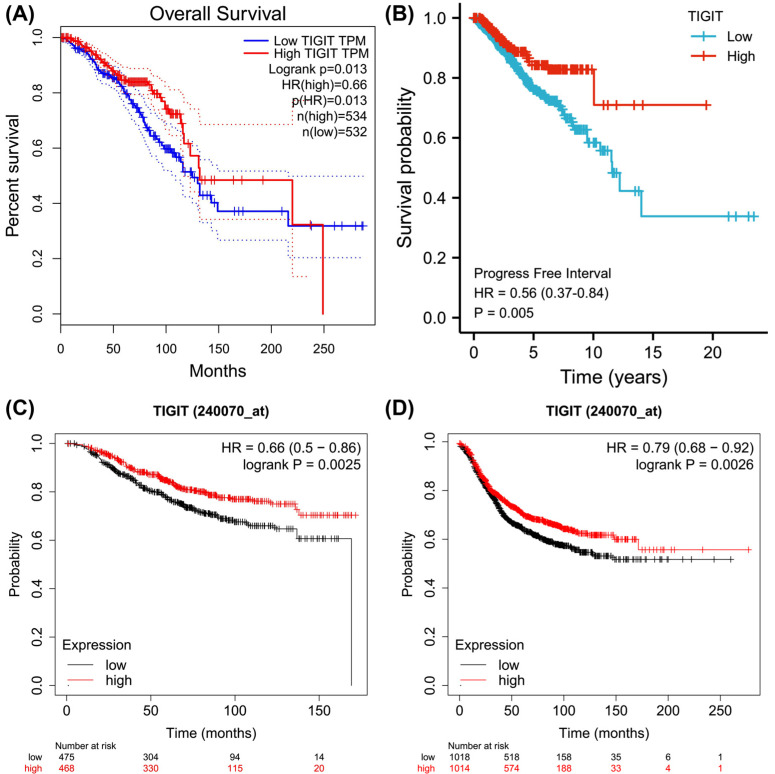
Kaplan Meier survival curve. (**A**) Overall Survival in TCGA; (**B**) Progressive Free Interval in TCGA; (**C**) Overall Survival in KM-plotter; (**D**) Relapse Free Survival in KM-plotter.

**Fig. (4):**
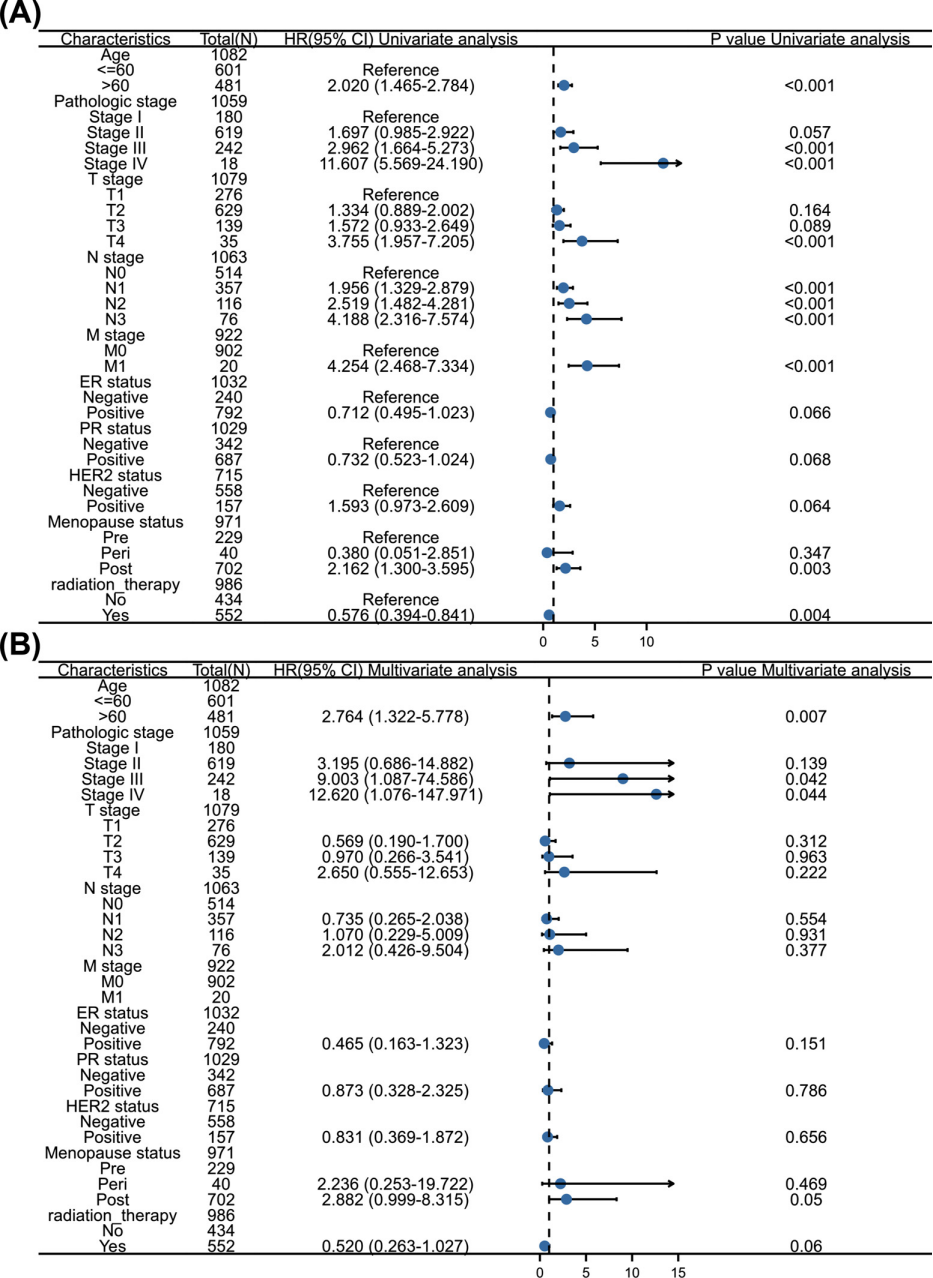
Univariate and multivariate regression analysis of clinicopathological factors related to OS in invasive breast cancer. (**A**) Forest map of single factor regression analysis; (**B**) Forest map of multivariate regression analysis.

Kaplan Meier survival analysis showed that TIGIT upregulation exhibited positive correlations with OS and PFI (*p=*<0.05, Figs. **3A** and **B**), which was consistent with results from KM-plotter database analysis (Figs. **3C** and **D**). With regards to the risk factors influencing the OS of invasive breast cancer patients, univariate analysis indicated that age, clinical stage, high TNM stage, menopausal status, and radiotherapy affect OS (*p=*<0.05), while multivariate analysis revealed that age, high clinical stage, and menopausal status were independent risk factors for tumor progression (*p=*<0.05, Fig. **4**).

### 3.4 TIGIT Upregulation has Diagnostic Value in Invasive Breast Cancer

**Fig. (5):**
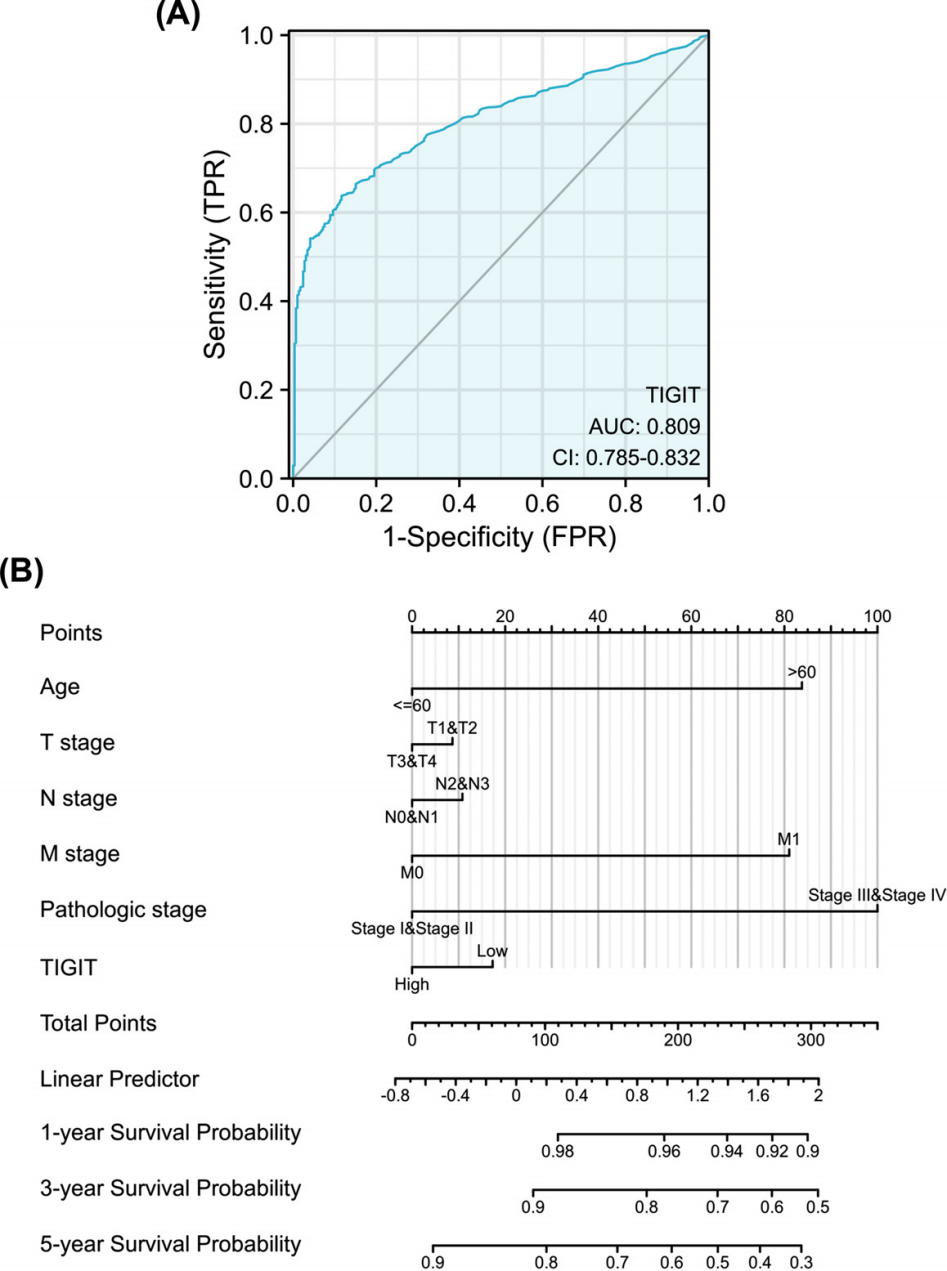
Diagnostic value of TIGIT expression in invasive breast cancer. (**A**) ROC survival curve; (**B**) Nomograms for predicting 1 -, 3 -, and 5-year overall survival.

To assess the diagnostic value of TIGIT, we performed an ROC curve analysis of TIGIT gene expression data. This analysis revealed an area under the ROC curve value of 0.809, implying that TIGIT can distinguish normal tissues from tumor tissues (Fig. **5A**). Next, we combined data on the TIGIT expression with clinical variables and constructed nomograms to predict 1-, 3-, and 5-year patient survival (Fig. **5B**).

### 3.5 PPI Networks and Enrichments

**Fig. (6):**
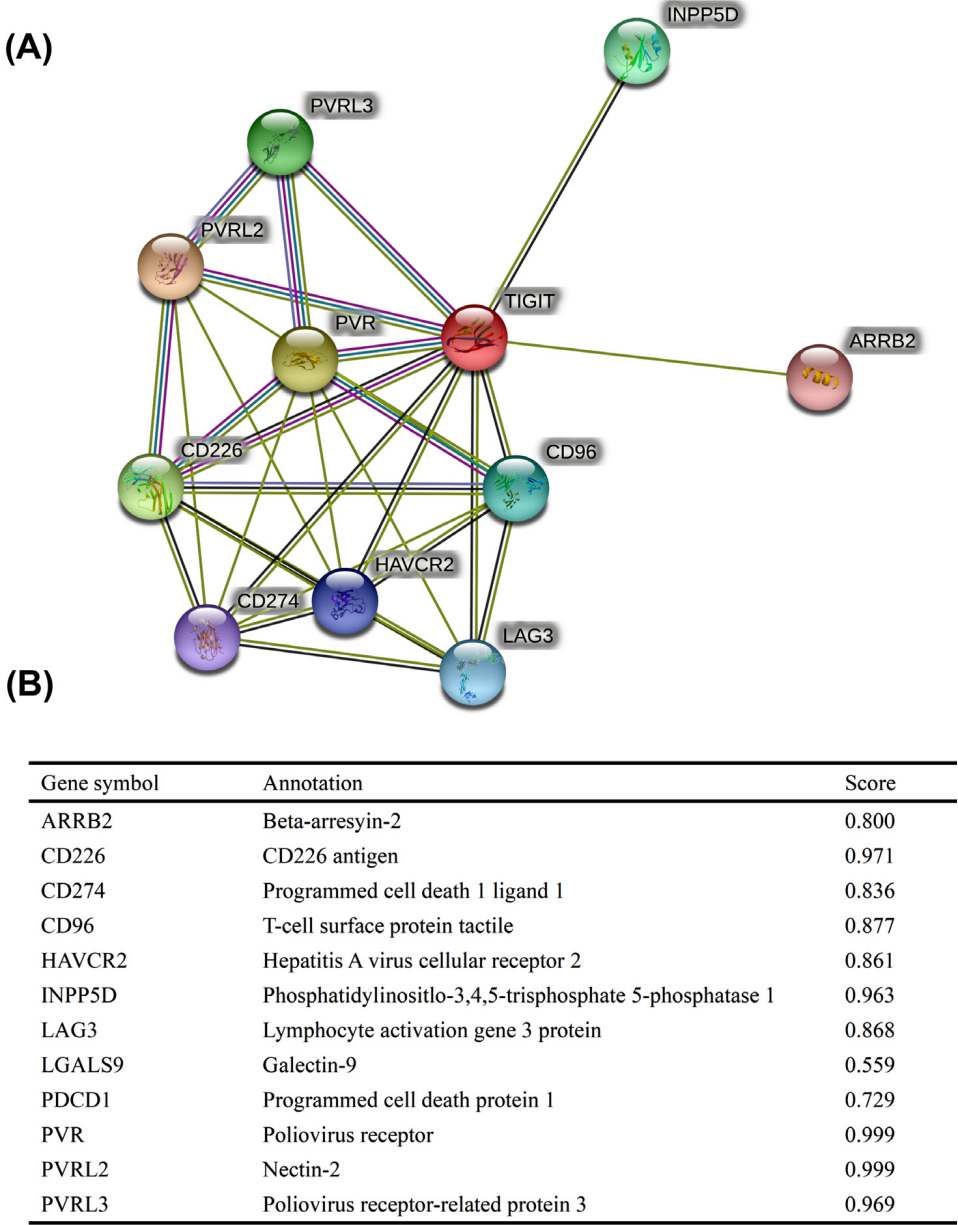
Protein-protein interaction comprehensive analysis of TIGIT. (**A**) HSPB1 network and its probable co-expression genes were evaluated by the STRING tool. The findings were visualized in a bubble chart; (**B**) Details of TIGIT-associated genes.

**Fig. (7):**
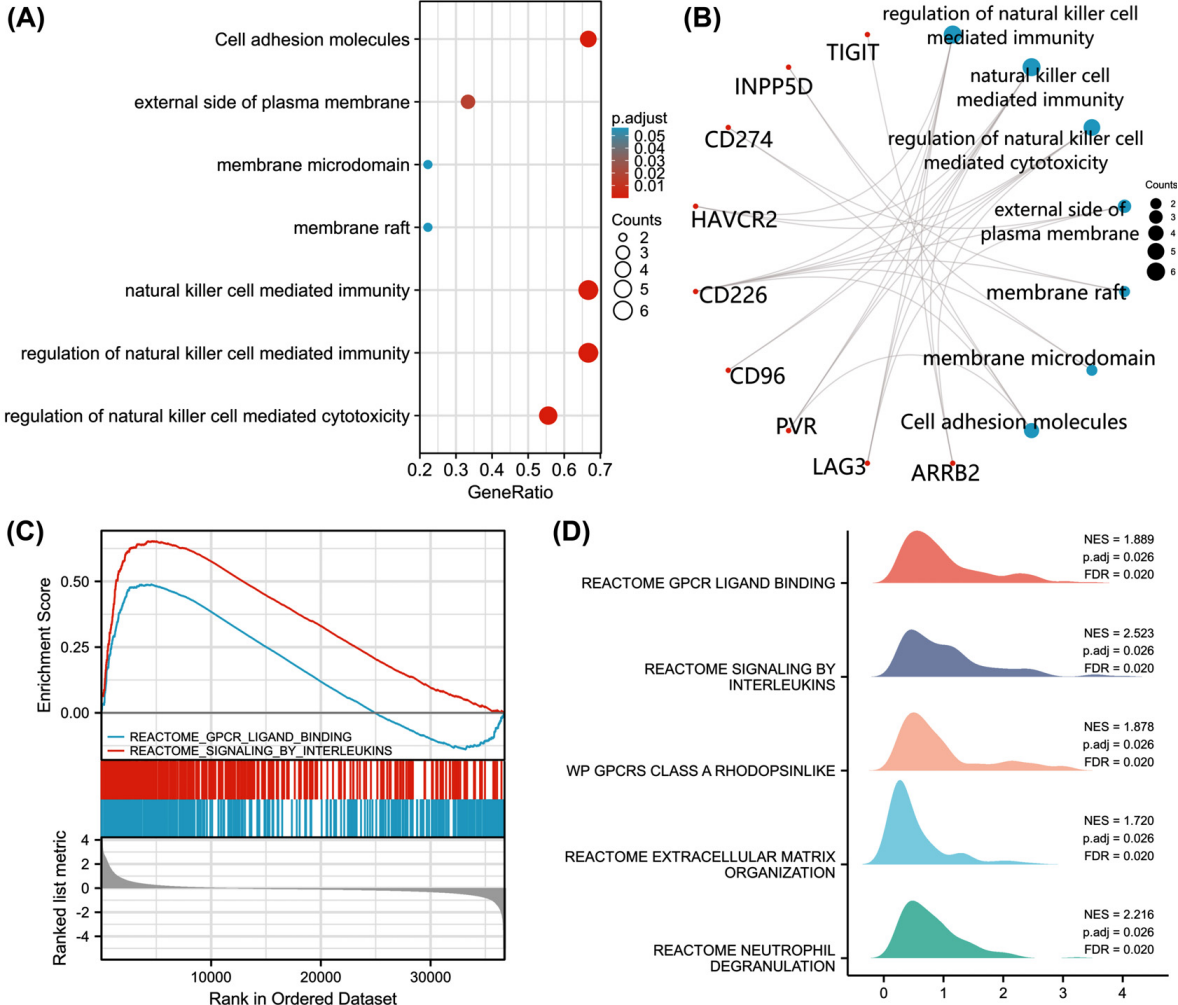
Enrichment analyses of TIGIT. (**A**) The bubble plot shows the signaling pathway of TIGIT; (**B**) TIGIT, INPP5D, CD274, CD226 and PVR were correlated in cell adhesion molecules; (**C-D**) enrichment map of GSEA gene set of reactome GPCR ligand binding and reactome siginaling by interleulcins in patients with TIGIT overexpression invasive breast cancer.

PPI network analysis using STRING identified various genes as being associated with TIGIT, including ARRB2, CD226, CD274, CD96, HAVCR2, INPP5D, LAG3, LGALS9, PDCD1, PVR, PVRL2, and PVRL3. Of these, CD226, INPP5D, PVR, PVRL2 and PVRL3 had interaction scores >0.96 (Fig. **6**). GO/KEGG enrichment analysis revealed that TIGIT, INPP5D, CD274, CD226, and PVR were enriched in cell adhesion molecules (Figs. **7A** and **B**). GSEA analysis identified reactome GPCR ligand binding and reactome signaling by interleukins as the most significantly enriched pathways in the high TIGIT-expression group (Figs. **7C** and **D**).

### 3.6 TIGIT was Significantly Correlated with Infiltrations of Immune Cells

**Fig. (8):**
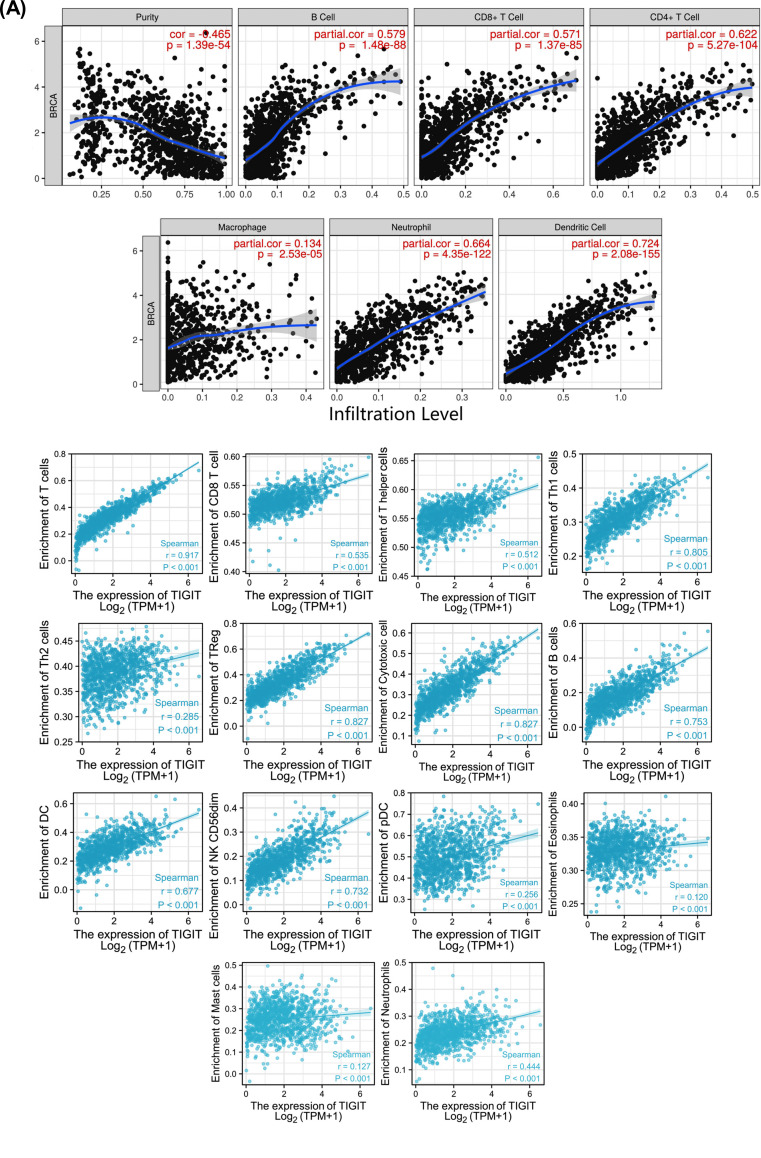
Association between TIGIT levels and immunocyte infiltrations in invasive breast cancer. (**A**) Relationship between TIGIT levels, tumor purity and six immune cell types was evaluated by the TIMER database. (**B**) Association between TIGIT and other immune cells.

Breast cancer is an immunogenic malignancy that is closely related to the immune environment. TIMER analysis suggests statistical significance between TIGIT and CD8^+^ T cells, B cells, CD4^+^ T cells, neutrophils, macrophages, and dendritic cells (*p=*<0.05, Fig. **8A**). Associations between TIGIT and other immunocytes were assessed by the GSVA package (Fig. **8B**).

## 4 DISCUSSION

TIGIT (T-cell immunoglobulin and ITIM domain protein) is a co-inhibitory transmembrane glycoprotein of the poliovirus receptor (PVR) family [13]. Studies indicate that TIGIT, PD-L1, and TIM-3 are significantly upregulated in the peripheral blood of patients with breast tumors [14]. TIGIT regulates the function of lymphocytes by interacting with the CD155 trans oligomer [10]. TIGIT has been implicated in the development of various tumors and is correlated with poor prognostic outcomes in various cancers, including colorectal, gastric, liver, melanoma, and head and neck squamous cell carcinomas [11, 15]. However, few studies have investigated its role in invasive breast cancer.

We performed a pan cancer analysis of TIGIT expression as well as its expression in invasive breast cancer using a TCGA dataset, and found that TIGIT was significantly upregulated in invasive breast tumor relative to normal tissues, and confirmed this observation using IHC. TIGIT mRNA levels were significantly different in age, T stage, ER level and PR level. We also found that TIGIT was highly expressed in basal-like and HER2-overexpressing subtypes of invasive breast cancer and used Kaplan-Meier survival analysis to verify the relationship between TIGIT and invasive breast cancer survival. Univariate and multivariate regression analyses of the relationship between TIGIT levels and clinic-pathological features associated with OS in invasive breast cancer revealed that age, high clinical stage, and menopausal status are independent risk factors for invasive breast cancer. Patients with high TIGIT levels had higher PFI and OS relative to those with low TIGIT expression.

Similar to PD-1, TIGIT is involved in inhibiting tumor directed immune responses. Both TIGIT and PD-1 are gradually upregulated in activated T lymphocytes, which are likely to inhibit excess immune reactions [16, 17]. TIGIT negatively regulates T cell activities by downregulating T cell receptor levels [16, 18]. In mice models and ongoing clinical research, blocking or ablating TIGIT, or blocking PD-1 alone or in combination, restores tumor inhibition [19, 20]. Mouse studies show that a combination of anti-PD-1 and anti-TIGIT markedly inhibits tumor growth, increases the ratio of cytotoxic T cells and regulatory T cells in tumors, and prolongs survival [21, 22]. Blocking or deleting TIGIT has been shown to promote NK cell-mediated antitumor responses and reduce the metastatic potential of tumor cells [22-24]. TIGIT combined with PD-1 blockade has been proved to promote tumor rejection in tumor model and enhance the antitumor effect of CD8^+^ T cells. This is a promising tumor immunotherapy, and these findings support the ongoing clinical trial of PD-1 / TIGIT double blocking in tumor patients [25, 26]. These studies, showing that TIGIT negatively modulates tumor immunity, are different from our findings. However, it is reported that TIGIT is protective and that it promotes liver regeneration by negatively regulating NK hepatocyte crosstalk [27]. An analysis of data from 1286 breast cancer samples found that TIGIT is associated with better breast cancer prognosis [28]. Taken together, these results suggest that TIGIT activity may differ across tumors and relative to normal tissues, and that it may have different roles due to cooperation with other immune molecules to regulate the immune microenvironment.

ROC curve analyses of the prognostic and diagnostic significance of TIGIT in invasive breast cancer revealed an area under the ROC curve value of 0.809, indicating diagnostic value. A nomogram constructed based on Cox regression analysis exhibited accuracy in the prediction of 1-, 3-, and 5-year survival rates of invasive breast cancer, and has practical significance for the development of clinical treatment. The nomogram’s c-index was 0.719 (95% CI: 0.694-0.743), indicating high accuracy.

PPI network analysis of TIGIT interactors found that the TIGIT related genes, INPP5D, CD274, CD226, and PVR were enriched in cell adhesion molecules. Because patients with high TIGIT expression had higher PFI and OS relative to those with low TIGIT levels, we evaluated its potential cellular mechanism using GSEA and found that in the high TIGIT group, reactome GPCR ligand binding, and reactome signaling by interleukins, were the most relevant enrichment pathways. Therefore, we will further explore this approach in future research in order to better explain this phenomenon.

Currently, few studies have investigated the relationship between TIGIT and immune cells in invasive breast cancer. Here, TIMER analysis of the association between TIGIT and infiltrations of immune cells showed that TIGIT was significantly different from CD8^+^ T cells, B cells, CD4^+^ T cells, neutrophils, macrophages, and dendritic cells. Invasive breast cancer markedly recruits tumor-infiltrating lymphocytes (TILs), particularly CD8^+^ T cells [29]. However, PD-1, TIGIT, and CTLA-4 are highly expressed in the TILs of TNBC patients, while PD-L1 and CD155 ligands are highly expressed in tumor cells or antigen presenting cells (APCs), which may make CD8^+^ T cells inefficient at killing tumor cells [30]. It is reported that the inhibitory receptor of CD155 is upregulated on the surface of effector lymphocytes during tumor progression, thereby inhibiting the cytotoxic killing ability of effector cells and inducing the immune escape of tumor cells [31].

In tumor immunity, T cells recognize tumor antigen by T cell receptor (TCR), initiate proliferation, activation and effect, and regulate the amplitude and quality of effect by the balance between costimulatory signal and inhibitory signal (Immune checkpoint). When exposed to the tumor microenvironment for a long time, the balance is broken and the inhibition signal is enhanced, leading to T cell depletion, and loss of proliferation, secretion of cytokines (including IL-2, TNF, and IFNγ), and degranulation by T cells. Depleted T cells are associated with high levels of various inhibitory receptors, such as TIGIT, CTLA-4, PD-1, and TIM-3 [32], which can reverse or at least partially reverse T cell depletion by blocking the inhibitory signaling pathway. T cell depletion is an important cause of immunosuppression and how to reverse tumor induced T cell depletion and reactivate cytotoxicity is a key question in immunotherapy. Our data indicate that TIGIT expression is closely related to breast cancer invasiveness. In addition to T cells, TIGIT can modulate anti-tumor immune responses by influencing other immune cells.

This study also has some limitations, including the small number of normal samples in the TCGA dataset, the small number of clinical samples validated by IHC, and the lack of in vivo validation using animal models. Thus, studies should be conducted to verify our findings.

## CONCLUSION

Our study shows that TIGIT expression is significantly upregulated in invasive breast cancer and that this correlates with patient prognosis. There are marked associations between TIGIT levels and infiltrations of immune cells. Therefore, our findings indicate that TIGIT levels may have a significant prognostic value in invasive breast cancer and that it may be a target for invasive breast cancer immunotherapy.

## Figures and Tables

**Table 1:** Clinical features of invasive breast cancer patients in TCGA.

**Characteristic**	**Low Expression of TIGIT**	**High Expression of TIGIT**	***p***
n	532	533	-
T stage, n (%)	-	-	0.004
T1	143 (13.5%)	132 (12.4%)	-
T2	283 (26.6%)	332 (31.3%)	-
T3	78 (7.3%)	59 (5.6%)	-
T4	25 (2.4%)	10 (0.9%)	-
N stage, n (%)	-	-	0.467
N0	259 (24.8%)	248 (23.7%)	-
N1	171 (16.3%)	178 (17%)	-
N2	50 (4.8%)	66 (6.3%)	-
N3	38 (3.6%)	36 (3.4%)	-
M stage, n (%)	-	-	0.236
M0	436 (48%)	453 (49.8%)	-
M1	13 (1.4%)	7 (0.8%)	-
Pathologic stage, n (%)	-	-	0.059
Stage I	103 (9.9%)	77 (7.4%)	-
Stage II	287 (27.5%)	319 (30.6%)	-
Stage III	118 (11.3%)	120 (11.5%)	-
Stage IV	12 (1.2%)	6 (0.6%)	-
Age, n (%)	-	-	0.013
<=60	273 (25.6%)	315 (29.6%)	-
>60	259 (24.3%)	218 (20.5%)	-
Histological type, n (%)	-	-	0.917
Infiltrating Ductal Carcinoma	369 (38.5%)	388 (40.5%)	-
Infiltrating Lobular Carcinoma	97 (10.1%)	105 (10.9%)	-
PR status, n (%)	-	-	< 0.001
Negative	138 (13.6%)	200 (19.7%)	-
Indeterminate	2 (0.2%)	2 (0.2%)	-
Positive	362 (35.6%)	312 (30.7%)	-
ER status, n (%)	-	-	< 0.001
Negative	79 (7.8%)	158 (15.5%)	-
Indeterminate	1 (0.1%)	1 (0.1%)	-
Positive	423 (41.6%)	355 (34.9%)	-
HER2 status, n (%)	-	-	0.388
Negative	264 (36.8%)	284 (39.6%)	-
Indeterminate	6 (0.8%)	6 (0.8%)	-
Positive	66 (9.2%)	91 (12.7%)	-
PAM50, n (%)	-	-	< 0.001
Normal	16 (1.5%)	24 (2.3%)	-
LumA	323 (30.3%)	228 (21.4%)	-
LumB	103 (9.7%)	99 (9.3%)	-
Her2	25 (2.3%)	57 (5.4%)	-
Basal	65 (6.1%)	125 (11.7%)	
Menopause status, n (%)	-	-	0.209
Pre	104 (10.9%)	120 (12.6%)	-
Peri	24 (2.5%)	15 (1.6%)	-
Post	345 (36.1%)	348 (36.4%)	-
Radiation_therapy, n (%)	-	-	0.158
No	227 (23.4%)	205 (21.1%)	-
Yes	258 (26.5%)	282 (29%)	-
OS event, n (%)	-	-	0.049
Alive	447 (42%)	471 (44.2%)	-
Dead	85 (8%)	62 (5.8%)	-
DSS event, n (%)	-	-	0.233
Alive	475 (45.4%)	490 (46.8%)	-
Dead	46 (4.4%)	35 (3.3%)	-
PFI event, n (%)	-	-	0.103
Alive	451 (42.3%)	471 (44.2%)	-
Dead	81 (7.6%)	62 (5.8%)	-

## Data Availability

The datasets used and/or analysed during the current study are available from the corresponding author [DWL] on reasonable request.

## References

[1] (2020). Guidelines for clinical diagnosis and treatment of advanced breast cancer in China (2020 Edition).. Chinese J. Oncol..

[2] Harbeck N., Penault-Llorca F., Cortes J., Gnant M., Houssami N., Poortmans P., Ruddy K., Tsang J., Cardoso F. (2019). Breast cancer.. Nat. Rev. Dis. Primers.

[3] Sung H., Ferlay J., Siegel R.L., Laversanne M., Soerjomataram I., Jemal A., Bray F. (2021). Global cancer statistics 2020: GLOBOCAN esti-mates of incidence and mortality worldwide for 36 cancers in 185 countries.. CA Cancer J. Clin..

[4] Bray F., Ferlay J., Soerjomataram I., Siegel R.L., Torre L.A., Jemal A. (2018). Global cancer statistics 2018: GLOBOCAN estimates of inci-dence and mortality worldwide for 36 cancers in 185 countries.. CA Cancer J. Clin..

[5] Adams S., Loi S., Toppmeyer D., Cescon D.W., De Laurentiis M., Nanda R., Winer E.P., Mukai H., Tamura K., Armstrong A., Liu M.C., Iwata H., Ryvo L., Wimberger P., Rugo H.S., Tan A.R., Jia L., Ding Y., Karantza V., Schmid P. (2019). Pembrolizumab monotherapy for previously untreated, PD-L1-positive, metastatic triple-negative breast cancer: Cohort B of the phase II KEYNOTE-086 study.. Ann. Oncol..

[6] Yu X., Harden K., Gonzalez L.C., Francesco M., Chiang E., Irving B., Tom I., Ivelja S., Refino C.J., Clark H., Eaton D., Grogan J.L. (2009). The surface protein TIGIT suppresses T cell activation by promoting the generation of mature immunoregulatory dendritic cells.. Nat. Immunol..

[7] Kong Y., Zhu L., Schell T.D., Zhang J., Claxton D.F., Ehmann W.C., Rybka W.B., George M.R., Zeng H., Zheng H. (2016). T-Cell immuno-globulin and ITIM domain (TIGIT) associates with CD8+ T-cell exhaustion and poor clinical outcome in aml patients.. Clin. Cancer Res..

[8] Johnston R.J., Comps-Agrar L., Hackney J., Yu X., Huseni M., Yang Y., Park S., Javinal V., Chiu H., Irving B., Eaton D.L., Grogan J.L. (2014). The immunoreceptor TIGIT regulates antitumor and antiviral CD8(+) T cell effector function.. Cancer Cell.

[9] Stengel K.F., Harden-Bowles K., Yu X., Rouge L., Yin J., Comps-Agrar L., Wiesmann C., Bazan J.F., Eaton D.L., Grogan J.L. (2012). Struc-ture of TIGIT immunoreceptor bound to poliovirus receptor reveals a cell-cell adhesion and signaling mechanism that requires cis-trans receptor clustering.. Proc. Natl. Acad. Sci. USA.

[10] Wu L., Mao L., Liu J.F., Chen L., Yu G.T., Yang L.L., Wu H., Bu L.L., Kulkarni A.B., Zhang W.F., Sun Z.J. (2019). Blockade of TIG-IT/CD155 signaling reverses t-cell exhaustion and enhances antitumor capability in head and neck squamous cell carcinoma.. Cancer Immunol. Res..

[11] He W., Zhang H., Han F., Chen X., Lin R., Wang W., Qiu H., Zhuang Z., Liao Q., Zhang W., Cai Q., Cui Y., Jiang W., Wang H., Ke Z. (2017). CD155T/TIGIT signaling regulates CD8+ T-cell metabolism and promotes tumor progression in human gastric cancer.. Cancer Res..

[12] Buyyounouski M., Choyke P., McKenney J., Sartor O., Sandler H., Amin M., Kattan M., Lin D. (2017). Prostate cancer - major changesin the American Joint Committee on Cancer eighth edition cancer staging manual.. CA Cancer J. Clin.

[13] Subramanian A., Tamayo P., Mootha V.K., Mukherjee S., Ebert B.L., Gillette M.A., Paulovich A., Pomeroy S.L., Golub T.R., Lander E.S., Mesirov J.P. (2005). Gene set enrichment analysis: A knowledge-based approach for interpreting genome-wide expression profiles.. Proc. Natl. Acad. Sci. USA.

[14] Dougall W.C., Kurtulus S., Smyth M.J., Anderson A.C. (2017). TIGIT and CD96: New checkpoint receptor targets for cancer immunotherapy.. Immunol. Rev..

[15] Elashi A.A., Sasidharan Nair V., Taha R.Z., Shaath H., Elkord E. (2018). DNA methylation of immune checkpoints in the peripheral blood of breast and colorectal cancer patients.. OncoImmunology.

[16] Zhang Q., Bi J., Zheng X., Chen Y., Wang H., Wu W., Wang Z., Wu Q., Peng H., Wei H., Sun R., Tian Z. (2018). Blockade of the check-point receptor TIGIT prevents NK cell exhaustion and elicits potent anti-tumor immunity.. Nat. Immunol..

[17] Zhang C., Wang Y., Xun X., Wang S., Xiang X., Hu S., Cheng Q., Guo J., Li Z., Zhu J. (2020). TIGIT can exert immunosuppressive effects on CD8+ T Cells by the CD155/TIGIT signaling pathway for hepatocellular carcinoma *in vitro.*. J. Immunother. (Hagerstown, Md. : 1997),.

[18] Liu X.G., Hou M., Liu Y. (2017). TIGIT, a novel therapeutic target for tumor immunotherapy.. Immunol. Invest..

[19] Manieri N.A., Chiang E.Y., Grogan J.L. (2017). TIGIT: A key inhibitor of the cancer immunity cycle.. Trends Immunol..

[20] Pauken K.E., Wherry E.J. (2014). TIGIT and CD226: Tipping the balance between costimulatory and coinhibitory molecules to augment the cancer immunotherapy toolkit.. Cancer Cell.

[21] Blake S.J., Dougall W.C., Miles J.J., Teng M.W., Smyth M.J. (2016). Molecular pathways: Targeting CD96 and TIGIT for cancer immunothera-py.. Clin. Cancer Res..

[22] Sarhan D., Cichocki F., Zhang B., Yingst A., Spellman S.R., Cooley S., Verneris M.R., Blazar B.R., Miller J.S., Adaptive N.K. (2016). Adap-tive NK cells with low tigit expression are inherently resistant to myeloid-derived suppressor cells.. Cancer Res..

[23] Xu F., Sunderland A., Zhou Y., Schulick R.D., Edil B.H., Zhu Y. (2017). Blockade of CD112R and TIGIT signaling sensitizes human natural killer cell functions.. Cancer Immunol. Immunother..

[24] Chan I.S., Knútsdóttir H., Ramakrishnan G., Padmanaban V., Warrier M., Ramirez J.C., Dunworth M., Zhang H., Jaffee E.M., Bader J.S., Ewald A.J. (2020). Cancer cells educate natural killer cells to a metastasis-promoting cell state.. J. Cell Biol..

[25] Chauvin J.M., Zarour H.M. (2020). TIGIT in cancer immunotherapy.. J. Immunother. Cancer.

[26] Ge Z., Peppelenbosch M.P., Sprengers D., Kwekkeboom J. (2021). TIGIT, the next step towards successful combination immune checkpoint therapy in cancer.. Front. Immunol..

[27] Bi J., Zheng X., Chen Y., Wei H., Sun R., Tian Z. (2014). TIGIT safeguards liver regeneration through regulating natural killer cell-hepatocyte crosstalk.. Hepatology.

[28] Fang J., Chen F., Liu D., Gu F., Chen Z., Wang Y. (2020). Prognostic value of immune checkpoint molecules in breast cancer.. Biosci. Rep..

[29] Budczies J., Bockmayr M., Denkert C., Klauschen F., Lennerz J.K., Györffy B., Dietel M., Loibl S., Weichert W., Stenzinger A. (2015). Classical pathology and mutational load of breast cancer - integration of two worlds.. J. Pathol. Clin. Res..

[30] Savas P., Virassamy B., Ye C., Salim A., Mintoff C.P., Caramia F., Salgado R., Byrne D.J., Teo Z.L., Dushyanthen S., Byrne A., Wein L., Luen S.J., Poliness C., Nightingale S.S., Skandarajah A.S., Gyorki D.E., Thornton C.M., Beavis P.A., Fox S.B., Darcy P.K., Speed T.P., Mackay L.K., Neeson P.J., Loi S. (2018). Publisher Correction: Single-cell profiling of breast cancer T cells reveals a tissue-resident memory subset associated with improved prognosis.. Nat. Med..

[31] Molfetta R., Zitti B., Lecce M., Milito N.D., Stabile H., Fionda C., Cippitelli M., Gismondi A., Santoni A., Paolini R. (2020). CD155: A mul-ti-functional molecule in tumor progression.. Int. J. Mol. Sci..

[32] Pauken K.E., Wherry E.J. (2015). Overcoming T cell exhaustion in infection and cancer.. Trends Immunol..

